# Patient iPSC-derived neurons reveal mechanisms underlying antidepressant response: a potential diagnostic tool

**DOI:** 10.1192/j.eurpsy.2023.274

**Published:** 2023-07-19

**Authors:** S. Shohat Koren, D. Kroitorou, C. Albeldas, A. Kugel, N. Askari, T. Cohen Solal, D. Laifenfeld

**Affiliations:** Genetika+, Tel Aviv-Yafo, Israel

## Abstract

**Introduction:**

Depression is a leading cause of disability worldwide despite dozens of approved antidepressants. There are currently no clear guidelines to assist the physician in their choice of drug, with existing tools limited to pharmacogenetics that have shown suboptimal response prediction outcomes resulting in a subscription process that is largely a trial and error one. Consequently, the majority of depressed patients do not respond to their first prescribed antidepressant, with >30% not responding to subsequent drugs. We report here on molecular readouts from an in vitro-based platform that provides patient-specific information on antidepressant mechanisms using cortical neurons derived individually from each patient.

**Objectives:**

To assess gene expression differences in prefrontal cortex neurons derived from responders and non-responders to two commonly used antidepressants, the selective serotonin reuptake inhibitor Citalopram and the atypical antidepressant Bupropion.

**Methods:**

Patient-derived lymphoblastoid cell lines from the Sequenced Treatment Alternatives to Relieve Depression (STARD) study with known response to Citalopram or Bupropion were reprogrammed and then differentiated to cortical neurons. Differential gene expression analysis was preformed to identify genes that are differentially expressed between drug responders and non-responders.

**Results:**

Significant differential expression was shown in 359 genes between Bupropion responders and non-responders (Fig1A) and 12 genes between Citalopram responders and non-responders (Fig1B). Clustering on the differentially expressed genes showed high agreement with the known response to both drugs (Fig1). Functional enrichment analysis revealed biologically relevant pathways that differ between responders and non-responders in Bupropion versus Citalopram.

**Image:**

**Figure 1.**
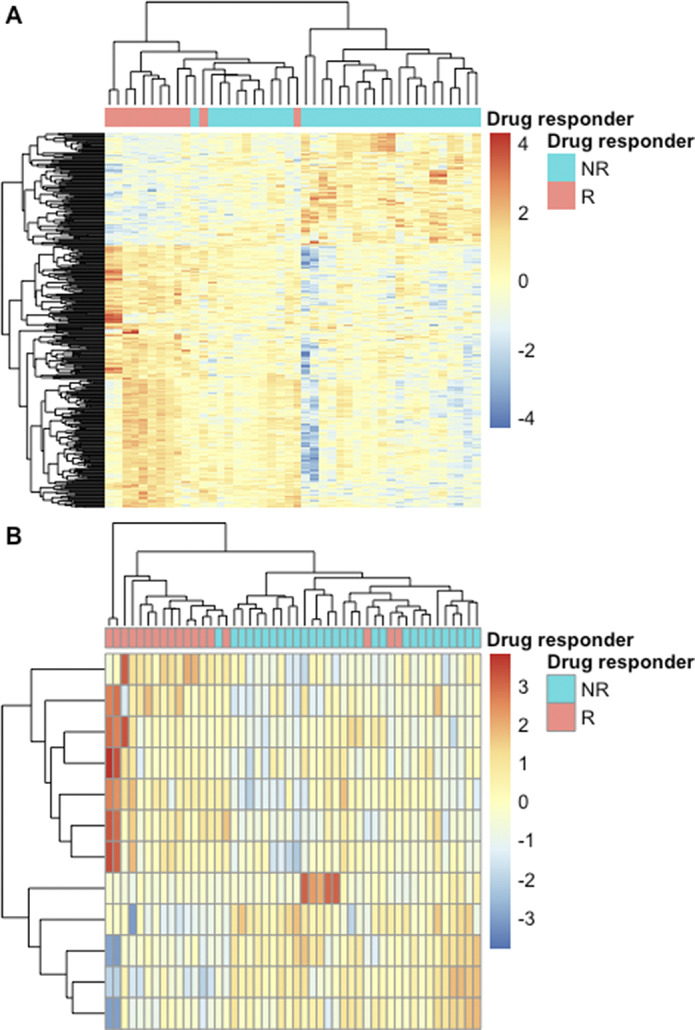
Heatmap of the expression of genes that show significant differential expression between neurons derived from Bupropion (A) and Citalopram (B) responders and non-responders. Color is the scaled gene expression; lines are genes and columns are samples. Column side colors represent the known response of the patient. Colum and line dendrograms are unsupervised hierarchical clustering.

**Conclusions:**

Gene expression patterns of neurons derived from patients with depression differ according to their response to two common antidepressants from different groups. The identification of distinct drug response dependent expression patterns in derived neurons can help elucidate mechanisms underlying antidepressant activity, supporting new drug development and response prediction.

**Disclosure of Interest:**

None Declared

